# Scotch Medical Schools

**Published:** 1891-09-12

**Authors:** 


### Scotch Medical Schools.

By a Scot.

It is difficult to convince a Scotchman that any Scotch institution is not, *ipso facto*, the best possible in the world, and to insinuate to a Scottish graduate any doubts as to the unquestioned superiority of the school that gave him his diploma is to invite immediate annihilation. The Scottish medical schools have, indeed, a well-earned fame, supported by their able and successful children in all parts of the earth. The manufacture of doctors is the staple industry of Edinburgh, but rich Glasgow and stubborn Aberdeen claim also to supply a large number of skilled healers to both the home and the foreign markets. The raw material with which each deals is different, and, therefore, certain differences mark the finished product, but Edinburgh, Glasgow, and Aberdeen are quite convinced that they can supply all the needs, social and professional, of the English-speaking race throughout the world, and are inclined to regard the existence of the London and English provincial schools as unnecessary, not to say impertinent.

Take the Aberdeen student, drawn from the hard and frugal north, ascetic in his habits, slow-speaking, thoughtful, incorrigibly earnest and persevering, capable of "plain living and high thinking" to an extent beyond the comprehension of more luxuriously reared men: he regards it as no grievance, but a privilege, to cultivate literature or science on a little oatmeal. Think of the advantage this gives him. A shrewd, though flippant, American writer recently remarked that in this age we could do many things; but we had forgotten how to do without. The Aberdonian scores here; he can do without a hundred things which most of us call necessaries. Therefore he can often climb to the top of the ladder, where others, hampered by their own desires, stop short half-way; or, if his fate so leads him, keep up in lonely districts, with hard and ill-paid work, and ideal of life and labour which prevents poverty and isolation from ever spelling defeat.

Or consider the Glasgow man, shrewd, keen, commercial rather than professional in instinct; not so much moved to devote himself to specialised study as to become a good all-round practitioner; fit to deal with the ordinary ills which flesh is heir to, and generally content to leave extraordinary ones to some one who has made particular study of them; the very man to deal with a club practice in a manufacturing town, or fill any other post where professional knowledge should be supplemented by insight into human nature with a special grasp of its possibilities of hypochondria and malingering.

The Edinburgh graduate thinks himself the *crême de la crême* of the profession. It has been impressed on him that to be a medical man, a spiritual descendant of the Hunters and Monroes, of Syme and Simpson, and Christison is to attain the highest rank that mortal man can win, and that the dignity of the profession is a thing he must unceasingly uphold. The feeling that *noblesse oblige* sustains him and restrains him when he is tempted to conduct which is traditionally regarded as unprofessional; to his own soul and conscience he is a traitor if he does anything that seems to dishonour his world-renowned and stately Alma Mater.

There are thus three different ideals in the three great Scottish universities, and being held and asseverated with that aggressive enthusiasm which is a national characteristic, they affect, as all earnestly felt convictions do, not only the native students, but the strangers within the gates of each palace of learning. The proportion of these varies in each place; and while in Glasgow and Aberdeen the Doric element predominates, Edinburgh is largely cosmopolitan. It is a magnetic mountain which draws men from all parts of the globe, allured by that fame which made it the greatest and keeps it the largest medical school in the world.

It is estimated that there are between three and four thousand medical students in Edinburgh, counting those who attend the extramural schools as well as those who belong to the university; and though the other towns cannot rival this, their schools are perhaps larger than any single one in England. In this lies their strength and their weakness. Exceptions there are and must be, but it is not generalising too rashly to say that for lectures a large school is the best, and for practical work a small one. The large class with the income arising from it, not merely helps to bring good men to fill a professional chair, but makes them independent of private practice, leaving the more time for preparation, study, and experiment. Good lectures are of fundamental importance to the student. Without them his text books, let him study them with the best will in the world, will prove too confusing for his untutored brain. It is the lecturer's function not merely to give him primary lessons in the subject he deals with him, but to guide him in his study of these books, to dwell upon cardinal points, yet give minor details their due importance. This he can do to a hundred pupils as easily as to one. If any of them fail to profit by his instructions the fault will lie in his own dulness and inattention, not in the size of the class. There are some experiments and demonstrations also which can easily be made clear to a considerable number; and in all such cases—assuming that the good lecturer is to be won by the good salary—a school like Edinburgh may claim to have the advantage.

But Edinburgh University must suffer from its size when practical work has to be considered. Many men prefer to do their dissecting in some of the extra-mural schools, where "parts" are more easily obtained, and the demonstrators can give them more individual attention. And there is no doubt that Edinburgh lacks clinical material. Large and splendid as its infirmary is it cannot supply the needs of its many students. "These poor Edinburgh men!" exclaimed the dean of a London school; "They work hard, and naturally feel aggrieved when they are plucked in their examination in London; but what can they learn of practical work when they have only one patient among three of them, and there is a clerk to do all the work?" That was no doubt an over-statement of the case. Hospital appointments in Edinburgh can be gained only by the few most brilliant students, and the large majority gain more working knowledge of their profession in six months as assistant to some general practitioner than they did in all their student career. They are happy only in having such an amount of theoretical knowledge as will enable them to comprehend easily the cases they meet with; and that before long they can successfully apply the principles they have learned.

Glasgow, on the other hand, has an admirable supply of clinical material. Its hospitals are filled not merely from the city itself but from a large district, the industries of which, with their accompanying accidents and diseases, are exceptionally varied. Hardly in London, Manchester, or Birmingham has a man greater opportunities of seeing every kind of case, both medical and surgical; and the "Second City" as it so proudly calls itself, may even claim a unique advantage in the amount of rickets to be found among its children. Among the teachers at the Glasgow medical schools, too, are honourable names, the names of men whose work is well known and highly esteemed, and the recent appointment of a lectureship in pathology makes its curriculum more complete than it has hitherto been. It is therefore the more to be regretted that the late scandal about the public health diploma at Glasgow University has robbed its diploma of some of its *prestige*. The action of a few too complaisant examiners has done a grievous wrong to many honourable men. Glasgow ought to be the seat of a great medical school, but it has driven from its doors many students who do not wish it to be thought that their diploma was too cheaply won.

Aberdeen has the advantage of moderate size and of a tradition of thoroughness in its work, but it might profit by some further development of the natural sciences in its curriculum. Its university is not rich, and it cannot boast such wealth of laboratory accommodation as Edinburgh and some other places. It is more adapted for turning out the general practitioner than the highly-developed specialist; but the product is good of its useful kind.

The Dundee Medical School has, or ought to have, a great future before it. It is seated in a wealthy and energetic town, that will be proud to transmute the gains of jute and marmalade into science and skill, and among its teachers are men of lofty aim and high accomplishment. It is too soon yet to speak of its work, but one may fairly hope that its student may give new renown to the diploma of "St. Andrew's by the northern sea."

